# Apical External Root Resorption and Repair in Orthodontic Tooth Movement: Biological Events

**DOI:** 10.1155/2016/4864195

**Published:** 2016-03-29

**Authors:** Liviu Feller, Razia A. G. Khammissa, George Thomadakis, Jeanine Fourie, Johan Lemmer

**Affiliations:** ^1^Department of Periodontology and Oral Medicine, Sefako Makgatho Health Sciences University, Pretoria 0204, South Africa; ^2^Private Practice, 15 School Road, Corner Rivonia Road, Morningside, Johannesburg, South Africa

## Abstract

Some degree of external root resorption is a frequent, unpredictable, and unavoidable consequence of orthodontic tooth movement mediated by odontoclasts/cementoclasts originating from circulating precursor cells in the periodontal ligament. Its pathogenesis involves mechanical forces initiating complex interactions between signalling pathways activated by various biological agents. Resorption of cementum is regulated by mechanisms similar to those controlling osteoclastogenesis and bone resorption. Following root resorption there is repair by cellular cementum, but factors mediating the transition from resorption to repair are not clear. In this paper we review some of the biological events associated with orthodontically induced external root resorption.

## 1. Introduction

Some apical external root resorption is an invariable side effect of orthodontic treatment. It affects most frequently the maxillary incisors and is associated with several biological and mechanical risk factors (Table 1) [1–11].

It is caused by orthodontic load-induced sterile inflammation that brings about resorption of the superficial root cementum, or it can become more severe with eventual resorption of the underlying dentin [10]. Apical external root resorption is ultimately repaired by cellular cementum but nevertheless may result in permanent loss of root length. Vital and endodontically treated teeth are equally affected [2], regardless of age [9].

Intrinsic factors that may play roles in the pathogenesis of orthodontic load-induced apical external root resorption include polymorphism of genes encoding cytokines and growth factors, alveolar bone density and turnover, hormonal deficiencies, and other local anatomical factors [4, 6, 12–16].

Reportedly, in about 80% of subjects, teeth undergoing orthodontic treatment may develop some degree of apical external root resorption [17]. It is almost impossible to establish any reliable estimate of the incidence, prevalence, or degree of severity of orthodontically induced apical external root resorption in terms of either subjects affected or teeth affected, because of differences in the published studies [18]. Studies have used small samples with different types, magnitudes, and durations of applied orthodontic forces. Single rooted and multirooted teeth at different stages of root development have been compared, as have been different orthodontic treatment techniques. Various methodologies with regard to selection criteria of teeth to be included in the studies and of evaluating the root resorption have been applied. Most studies have been retrospective and nonrandomized with orthodontic treatment of different durations, and not all have taken into account systemic or local risk factors [3, 18–21].

Many studies have been carried out on laboratory animals, and the results of such studies, although providing important information on the pathogenic mechanisms of apical external root resorption, cannot be reliably extrapolated to the outcome of treatment of patients [18], nor can studies investigating external root resorption in relation to the number of treated patients be compared to studies investigating the proportion of orthodontically treated teeth which were affected by apical external root resorption [17].

Nevertheless, it is estimated that up to 90% of orthodontically treated teeth have some degree of apical external root resorption, and up to 15% of these cases show severe apical resorption of more than 4 mm (Table 2) [5, 7, 17]. However, in the vast majority of cases, the reduction in root length is slight and clinically insignificant and does not affect the prognosis of the involved teeth [18, 22, 23]. Root resorption that occurs during orthodontic tooth movement ceases at the end of treatment; but in fact, some repair with cellular cementum occurs [24, 25].

Since the area of periodontal attachment of a tapering root per millimeter of root length decreases very substantially from the cervical towards the apical region, it is estimated that the loss of periodontal attachment from 3 mm of apical external root resorption is equivalent to about 1 mm of cervical bone loss [22]. Indeed, even following severe apical external root resorption of more than 4 mm, the area of periodontal attachment loss is such that the teeth will continue to function normally for many years [24].

Cementum is the thin layer of mineralized tissue at the root surface providing anchorage for the principal fibers of the periodontal ligament [26, 27]. Cementoblasts, like osteoblasts, express the transcription factors runt-related gene 2 (Runx 2) and osterix which transactivate genes encoding for type 1 collagen, alkaline phosphatase (ALP), bone sialoprotein (BSP), and osteocalcin. It appears that osterix has an important role in regulating the formation of both cellular cementum and bone, by downregulating cell proliferation and upregulating cell differentiation and mineralization [28].

Despite the fact that the differentiation of both cementoblasts and osteoblasts is driven by bone morphogenetic proteins (BMPs), it is unclear whether cementoblasts and osteoblasts are derived from a common mesenchymal multipotential precursor cell [26, 27] or whether cementoblasts originate from an epithelial progenitor cell through epithelial-mesenchymal transition [28] or whether a common cell type mediates the formation of the different subtypes of cementum [29]. In any case it has been suggested that both osteoblasts forming bone and cementoblasts forming cellular cementum arise from a common progenitor cell [29, 30].

While in many aspects cementum is similar to bone, cementum differs from bone in having only a limited capacity for remodelling, in lacking innervation, vascularization, or lamellar structure, and in lacking any role in calcium homeostasis or haemopoiesis [19, 26, 30–32]; and the lacunocanalicular network is less developed in cementum than in bone [29, 31].

## 2. Physiological “Remodelling” of Cementum

Cementum resorption and subsequent repair by cellular cementum (cementum turnover), which according to some researchers can be regarded as a physiological remodelling process, occurs throughout life in response to metabolic changes in the periodontal ligament mediated by forces of mastication, by continual tooth eruption and drifting, and possibly by parafunctional gnathic activities [10, 32–34]. On the other hand, other researchers are of the opinion that, unlike bone, cementum does not undergo such physiological remodelling but increases in thickness throughout life [30, 35]. Regardless of this debate, external dentin resorption will not occur unless there has been a full-thickness cemental resorption [30, 32, 36, 37].

Resorbed acellular cementum is always repaired with cellular cementum but the molecular pathways and cellular mechanisms which mediate this process are unknown [30, 33]. External root resorption may be induced not only by forces exceeding the physiological limits but also by intrinsic alterations in the Wnt/*β*-catenin and in the receptor activator of nuclear factor *κβ* (RANK), RANK ligand (RANKL), and osteoprotegerin (OPG) signalling pathways. Under these circumstances, the extent of the resorption is influenced by the degree of dysregulation of these pathways [12].

## 3. Apical External Root Resorption during Orthodontic Treatment

The site of orthodontically mediated resorption on a root surface is determined primarily by whether the tooth is moved bodily or is tipped and occurs predominantly on those parts of the root surfaces exposed to high compressive stresses [11]; but it can also occur, though to a lesser extent, on root surfaces exposed to tensile stresses within the periodontal ligament [19]. The greater the compressive stress is, the greater the root resorption will be [19].

Bodily tooth movement generates compressive stresses along root surfaces with resorption of cementum at zones of compression of the periodontal ligament, but such resorption is less frequent and less severe than the apical root resorption associated with tipping tooth movements [11] because with tipping movements, compressive stresses are concentrated at the thin apical portion of the root where not only is the tooth movement greater, but also the stress per unit surface area is more than that at the much thicker cervical portion of the root [19, 38, 39].

Furthermore, the thinner, more elastic alveolar bone around the cervical one-third of the root has the capacity to absorb much more of the orthodontically induced mechanical stress compared to the thick, less elastic alveolar bone that envelops the apical one-third of the root [8, 21, 38, 39].

Orthodontically induced root resorption can be evaluated by conventional radiography, subtraction radiography, cone-beam computed tomography, scanning electron microscopy, and histopathology [11, 20]. Cone-beam computed tomography is the only method that provides three-dimensional information about root resorption in a clinical setting, while periapical or panoramic radiography is two-dimensional and is subject to magnification errors and to low observer reproducibility [11, 40]. Histopathological examination and scanning electron microscopy are clearly not clinically applicable [11].

In general, in both bodily and tipping tooth movements, external root resorption increases with the magnitude of the applied orthodontic force [3, 14, 19, 41] and with continuous forces [3, 39, 42], and these factors can be controlled by the type of orthodontic appliance used [8, 39]. It seems that there is less external root resorption when interrupted or intermittent orthodontic forces are applied because cementum repair can occur in the intervals between force applications [3, 10, 33].

## 4. Resorption of Cementum in the Context of Orthodontic Tooth Movement

In the context of orthodontic tooth movement, the necrosis and hyalinization in the periodontal ligament and alveolar bone occur in response to localized orthodontic load-induced compression of the vasculature and subsequent ischaemia in the periodontal ligament and adjacent alveolar bone [19, 37, 43]. Subsequently, removal of necrotic and hyalinized tissues in the compressive zone of the periodontium by macrophage-like cells, multinucleated cells, osteoclasts, and cementoclasts/odontoclasts has the collateral damaging effect of external root resorption [44–46]. However, it is also possible that direct damage to the superficial layer of the cementum is caused by clast cells-mediated cementum resorption, without necrosis and hyalinization in the periodontal tissues [1].

During load-induced resorption of cementum, it appears that, as in the case of osteoclast differentiation and function [43], a number of biological mediators, including hormones, cytokines, macrophage colony-stimulating factor (M-CSF), and RANK, RANKL, and OPG signalling pathway, play important regulatory roles in the differentiation and function of cementoclasts/odontoclasts [47–49]. The expression of RANKL is upregulated by fibroblasts, osteoblasts, and other resident and transient cells in the compressed periodontal ligament in response to orthodontic loading [49].

Cementoclasts/odontoclasts have a phenotype similar to that of osteoclasts [15], being multinucleated and having ruffled borders, clear zones, abundant mitochondria, rough endoplasmic reticulum, and tartrate-resistant acid phosphatase (TRAP) activity; but in contrast to osteoclasts, the origin of these cells and the molecular pathways that drive their differentiation are not well understood [50, 51]. However, it appears that precursors of cementoclast/odontoclasts originate from circulatory mononuclear cells that extravasate in the periodontal ligament in response to chemotactic agents and then migrate to the areas of resorption on the root surface [51] and that their resorptive activity is also controlled by the RANK/RANKL/OPG signalling pathway expressed in the periodontal ligament [49].

## 5. Repair of Cementum in the Context of Orthodontic Tooth Movement

After the cementoclasts/odontoclasts have become detached from the resorption lacunae, early cementum repair starts with fibroblast-like cells from the periodontal ligament invading the resorption lacunae [44]. These fibroblast-like cementoblastic cells secrete noncollagenous matrix proteins, particularly osteopontin and bone sialoprotein, filling the spaces in the residual collagen fibril structure [44]. Thus, the architecture of the collagen structure determines the pattern of deposition of the noncollagenous proteins [35]. Subsequently, the cementoblasts secrete collagen fibrils that intermingle with the residual fibrils of the existing collagenous structure forming a thin cementoid repair matrix [44–46]. Mineralization ensues with hydroxyapatite crystals development and growth between the collagen fibrils [35], forming reparative cementum of the cellular intrinsic fiber type [25, 44].

During this process of cementum repair, extracellular matrix proteins from resorbed cementum including fibronectin, osteopontin, and osteocalcin contribute to the recruitment of cementoblast precursors to the root surface and to their subsequent adhesion, proliferation, and differentiation. Local cytokines and growth factors including insulin-like growth factor- (IGF-) 1, fibroblast growth factor (FGF), epidermal growth factor (EGF), bone morphogenetic proteins (BMPs), and transforming growth factor-*β* (TGF-*β*) also play important roles in cementoblast precursor differentiation and cementoblast proliferation, further promoting cementogenesis [44].

As mentioned previously, epithelial cell rests of Malassez in the periodontal ligament may also play some role in the formation of reparative cementum [28, 52]. Upregulation of the Wnt/*β*-catenin signalling pathway can promote epithelial-mesenchymal transition with dedifferentiation of the epithelial cell rests of Malassez which subsequently differentiate into cementoblast-like mesenchymal cells that can contribute to the formation of reparative cementum [28, 52]. It is also possible that epithelial cell rests of Malassez promote cementum formation indirectly by secreting inductive signalling mediators [52].

The Wnt/*β*-catenin signalling pathway, in addition to its role in epithelial-mesenchymal transition in the context of cementogenesis, is essential for osteoblast differentiation during embryogenesis and postnatally for regulating osteoblast proliferation. Canonical Wnt/*β*-catenin signalling is characterized by accumulation of *β*-catenin in the cytoplasm and subsequently its translocation into the nucleus of both osteoblasts and cementoblasts where it interacts with members of the Lef/Tcf family of transcription factors [53].

Wnt/*β*-catenin signalling is regulated by a balance between agonists of frizzled receptors and LRP 5/6 coreceptors on one hand and several antagonists including Dkks and sclerostin on the other hand [53]. In addition, upregulated levels of *β*-catenin in mature osteoblasts may induce the production of osteoprotegerin resulting in inhibition of differentiation and of functional activity of osteoclasts. Thus, the canonical Wnt/*β*-catenin signalling pathway via different vectors plays an essential role in bone turnover and subsequently in the regulation of bone mass [53].

Runx 2 is the master transcription factor that upregulates the genes ALP, collagen type 1, osteocalcin, and osteopontin that mediate osteogenic differentiation [54]. Runx 2 is expressed not only by osteogenic cells, but also by the cells laying down reparative cementum within the resorption lacunae [44]. Osterix is another transcription factor essential for osteogenesis and cementogenesis, and like Runx 2, it positively regulates the expression of genes encoding osteopontin, osteocalcin, and bone sialoprotein and the levels of their encoded proteins [27, 55].

In relation to cementogenesis, the activated Wnt/*β*-catenin signalling pathway in cementoblasts inhibits the expression of Runx 2 and osterix, resulting in downregulation of cementoblast differentiation and proliferation [26]. Conversely, downregulation of the Wnt/*β*-catenin signalling pathway is positively associated with differentiation of cementoblasts and subsequent increased formation of cementum [27]. However, other researchers have reported that, on the contrary, upregulation of the Wnt/*β*-catenin signalling is associated with increased cementum formation [56]; but downregulated Wnt/*β*-catenin signalling is associated with increased resorption of cementum [12]. In any event, the modulation of the Wnt/*β*-catenin signalling pathway plays an important role in the differentiation of cementoblasts and in the formation of cementum [12, 57].

Osterix is a transcription factor essential for the differentiation of preosteoblasts and precementoblasts into mature osteoblasts and cementoblasts and functions molecularly downstream from Runx 2. By inhibiting the Wnt/*β*-catenin signalling in cementoblasts and osteoblasts, probably via upregulation of DKK1 expression, osterix inhibits osteoblast and cementoblast proliferation [27, 28, 53].

Sclerostin is a glycoprotein encoded by the SOST gene, expressed by terminally differentiated cells within mineralized matrices, regulating osteoblast and cementoblast proliferation and differentiation, and ultimately playing an important role in bone formation, maturation, and remodelling [29, 58]. Sclerostin is an inhibitor of the canonical Wnt/*β*-catenin signalling pathway and of pathways of several members of BMP family. These pathways are essential for driving the process of commitment and subsequent differentiation of multipotential mesenchymal progenitor cells along the osteoblastic and cementoblastic lineages [29, 31, 59]. Whereas downregulation of sclerostin in osteocytes may cause sclerosteosis, the effect of such downregulation on cementum is unclear [58].

Orthodontic load-generated strains on cementoblast precursors in the periodontal ligament induce Cox-2 mRNA expression and the synthesis of prostaglandin (PGE_2_). Subsequently, PGE_2_ may mediate differentiation of cementoblasts and upregulate the expression of genes involved in mineral metabolism, including osteocalcin and alkaline phosphatase [36].

BMPs, members of the transforming growth factor-*β* (TGF-*β*) superfamily, are found in high concentration in mineralized tissues. BMPs transduce intracellular signals through Smad-dependent molecular pathways and thorough Smad-independent molecular pathways, interacting with various transcription factors and regulating the expression of target genes [60]. BMPs upregulate the expression of Runx 2 and both independently and cooperatively stimulate osteogenic and cementogenic gene expression [55]. The BMP signalling cross-talks with the Wnt/*β*-catenin signalling pathway, and depending on the tissue involved, these pathways may act synergistically or antagonistically [60]. On the other hand, BMPs have the capacity to induce cementogenesis with cementoblast-generated extracellular matrix formation, mineralization, and the regeneration of Sharpey fibers [55, 60, 61].

BMP-2 promotes differentiation of cementoblast progenitor cells into a cementogenic lineage but has little mineralization-inducing effect on mature cementoblasts. Thus, any mineralization-promoting effect of BMP-2 is probably indirect through its interaction with progenitor cells, mediating the increase in the pool of mature cementoblasts [59, 62]. On the other hand, BMP signalling pathways in cementoblasts have the capacity to downregulate Wnt/*β*-catenin signalling by activating the Wnt/*β*-catenin inhibitors Dkk1 and sclerostin [52, 57].

## 6. Clinical Considerations in relation to Orthodontic Load-Induced External Root Resorption

As previously mentioned, strategies to minimize external root resorption should include identification of systemic and local risk factors at the stage of treatment planning, limitation of treatment duration, the use of light intermittent forces, and biannual radiographic monitoring in order to detect any possible root resorption at the earliest stage [63]. As some degree of apical external root resorption is a frequent and unavoidable complication of orthodontic treatment, during treatment planning, the patient or parent should be warned of this risk.

It appears that if irregularities of root contour indicative of apical external root resorption are not detected within six to nine months of commencement of orthodontic treatment, then it would be unlikely that any significant apical external root resorption will occur, but if any apical external root resorption is observed within six to nine months from the beginning of orthodontic treatment, then there is a high risk of further root resorption [64, 65].

If any apical external root resorption is detected, active treatment should be suspended for two to three months, hopefully to prevent further resorption and to allow some healing with cellular cementum. If further resorption is detected after active treatment has been resumed, the orthodontic treatment plan should be modified [6].

## Figures and Tables

**Table 1:** Risk factors-associated with orthodontic treatment-induced apical external root resorption [1–11].

(1) Genetic factors	
(2) Systemic factors	
(3) Personal susceptibility	
(4) Root morphology	
(5) Alveolar bone morphology	
(6) The magnitude, type (continuous or intermittent), direction and duration of the applied orthodontic force	
(7) Type of treatment mechanics (rectangular or round arch wires, springs, elastics, etc)	
(8) Nature of tooth movement (intrusion, extrusion, tipping or bodily movement)	
(9) Distance of tooth movement	
(10) Overall duration of orthodontic treatment	

**Table 2:** Classification of degree of external root resorption (based on [4, 7, 23, 24]).

Mild	Apical root resorption less than 2 mm of the original root length

Moderate	Apical root resorption greater than 2 mm but less than one-third of the original root length

Severe	Root resorption exceeding 4 mm or one-third of the original root length

## Additional Points

Some degree of load-induced apical external root resorption is a frequent and an unavoidable complication of orthodontic treatment. The process of external root resorption is complex involving interplay between the various molecular signalling pathways that drive the resorption of cementum and dentin by odontoclasts/cementoclasts. The occurrence of such root resorption will be minimized by careful planning, execution of treatment, and radiographic monitoring.
